# Methodology to Quantify and Screen the Demineralization of Teeth by Immersing Them in Acidic Drinks (Orange Juice, Coca-Cola™, and Grape Juice): Evaluation by ICP OES

**DOI:** 10.3390/molecules26113337

**Published:** 2021-06-01

**Authors:** Eliane S. P. Melo, Elaine Melo, Daniela Arakaki, Flavio Michels, Valter A. Nascimento

**Affiliations:** 1 Group of Spectroscopy and Bioinformatics Applied Biodiversity and Health (GEBABS), Post-Graduate Program in Health and Development in the Midwest Region, School of Medicine, Federal University of Mato Grosso do Sul, Campo Grande 79070-900, Brazil; elianedepadua60@gmail.com (E.S.P.M.); elaine.melo@ufms.br (E.M.); daniarakaki@gmail.com (D.A.); 2 Physics Institute, Federal University of Mato Grosso do Sul, Campo Grande 79070-900, Brazil; flavio.michels@ufms.br

**Keywords:** demineralization, ICP OES, minerals, erosive challenge, pH

## Abstract

Oral health problems may occur as a result of the ingestion of acid drinks. The objective of this in vitro study was to quantify and screen the concentration of potassium (K), phosphorus (P), calcium (Ca), magnesium (Mg), manganese (Mn), zinc (Zn), iron (Fe), copper (Cu), barium (Ba), lead (Pb), arsenic (As), cadmium (Cd), aluminum (Al), cobalt (Co), chromium (Cr), molybdenum (Mo), sodium (Na), nickel (Ni), selenium (Se), and vanadium (V) released from bovine incisors during an erosive challenge at different times of exposure when immersed in Coca-Cola™, orange juice, and grape juice. A total of 240 samples of bovine incisor teeth were used for the erosive challenge and allocated in groups. Digestion of drinks was performed using microwave-assisted digestion. The content in acidic drinks was monitored before and after the erosive challenge at exposure times of 1, 5, and 60 min using inductively coupled plasma optical emission spectrometry (ICP OES). The drinks’ pH varied slightly during the erosive challenge but remained below the critical value of pH 5 to cause tooth demineralization. The concentrations of elements released from the bovine incisors during the in vitro erosive challenge depend on exposure times when immersed in acidic beverages. For some elements such as Ca, Mn, Zn, Fe, Cu, Ba, Pb, As, and Cd, quantified in acidic drinks, grape juice had greater erosive potential than Coca-Cola™ and orange juice. Quantification and monitoring of chemical elements in bovine teeth can be performed considering a longer erosive time and other types of acidic drinks. Further analysis using human teeth is still not available and must be conducted. The demineralization of teeth not only occurs in acidic beverages; physical and chemical factors play other roles and should be investigated.

## 1. Introduction

The demineralization and the subsequent erosion and/or loss of the tooth surface have become the target of studies and concerns for dental science, the dental profession, and the patient [1,2,3]. Tooth erosion can be caused by medication, lifestyle factors, diet, gastric acid, vomiting or gastroesophageal reflux disease, and fruit-flavored beverages [4,5,6,7,8]. Also, various eating habits for a short or long period can cause tooth erosion [9,10]. Acidic drinks are the ones that most cause erosive injuries [3,4].

There are several opinions about pH’s value and its erosive power [2,3,5,9]. According to studies, the higher the drink’s titratable acidity, the greater its erosive power over dental structures [2,3]. Some publications also believe that a pH above 5.0 is not significant for dental erosion [11]. On the other hand, some researchers consider other important parameters such as acid concentration, degree of saturation, calcium and phosphate concentrations, and erosion inhibitors to influence tooth erosion [12].

Contrary to studies [2,3,11,12], some researchers combined the erosion test with the exposure time (days) to analyze the wear of the enamel using contact profilometry [13]; additionally, some studies have focused on the loss of enamel and dentin weight after days of exposure [14], as well as measuring calcium loss by atomic absorption spectroscopy [11]. Beltrame et al. [15] evaluated grape and orange juices’ chemical characteristics and their erosive potential to reduce microhardness and enamel structure loss.

In a recent paper, the elements Fe, Cd, K, S, Co, Mg, Mn, Zn, Al, and Cu were quantified across different human teeth types using inductively coupled plasma resonance mass spectrometry (ICP MS). However, they did not consider exposure time in beverages [16]. In fact, without considering the exposure time of teeth in beverages, previous studies have only quantified macro- and micro-elements in teeth using ICP MS and inductively coupled plasma atomic emission spectroscopy (ICP OES) [17], atomic absorption spectroscopy (AAS), ICP MS [18,19], and inductively coupled plasma-atomic emission spectrophotometry analysis (ICP AES) [20].

Although Jager et al. [11] analyzed the loss of calcium before and after immersion of bovine teeth in various types of drinks monitoring the exposure period, other elements were not quantified in their study. Elements such as Al, Cd, Co, Cu, Fe, K, Mg, Mn, S, and Zn may be present in the human enamel and dentin [16]. Riyat and Sharma [20] placed human teeth in nitric acid for two days until complete dissolution, and quantified thirty-four elements (Si, Al, Fe, Ca, Mg, K, Mn, Ti, P, Li, Be, B, V, Cr, Co, Ni, Cu, Zn, As, Sr, Y, Nb, Mo, silver Ag, Cd, tin Sn, Sb, Ba, La, Ce, W, Pb, Bi, and Zr) by ICP AES. Thermogravimetric analysis coupled to mass spectrometry (TG-MS) and wavelength dispersive X-ray fluorescence (WDXRF) showed that human and bovine enamel and dentine present the maximum similarity among the species analyzed [21]. Bovine dentine and enamel presented the most similar C, Na, Mg, P, S, Cl, K, Ca, Fe, Cu, Zn, Sr and Ca/P values to human dentine enamel [21]. Besides, the Ca/P ratio of the mineral removed from the enamel surfaces during demineralization and remineralization is the same in both human and bovine enamel [22]. Although there are several studies on the erosive challenge [11,12,13,23,24], there is a lack of studies quantifying the concentration of elements such as K, P, Ca, Mg, Mn, Zn, Fe, Cu, Ba, Pb, As, Cd, Al, Co, Cr, Mo, Na, Ni, Se and V in human or bovine teeth when immersed in diverse types of drinks and at different times of exposure. The process of tooth mineralization and demineralization in acidic beverages involving various macro- and micro-elements and their behavior as a function of exposure time are missing.

Motivated by the paper published by Jager et al. [11], Sharma et al. [16], and Riyat and Sharma [20], in this study, a methodology was developed to quantify and screen the concentration of K, P, Ca, Mg, Mn, Zn, Fe, Cu, Ba, Pb, As, Cd, Al, Co, Cr, Mo, Na, Ni, Se, and V released from bovine incisors during the in vitro erosive challenge at different times of exposure when immersed in Coca-Cola™, orange juice, and grape juice. In addition, a microwave-assisted digestion optimization methodology was employed to quantify minerals from the demineralization of teeth. The analytical technique ICP OES was used to determine elements in the samples of drinks.

## 2. Results

### 2.1. pH Monitoring

As shown in Table 1, the pH value of Coca-Cola™, grape juice, and orange slightly varied during the erosive challenge (t = 0, 1, 5, 30, and 60 min). Table 1 contains the results of the measured temperatures of the drinks before and for each exposure time. The pH of Coca-Cola™ and grape juice drinks changed with exposure time, while the pH of orange juice decreased (Table 1). In addition, the pH of ultrapure water remained constant during the exposure time.

### 2.2. Accuracy of the ICP OES

A critical quantification in chemical analysis is the determination of detection limit (LOD), limit of quantification (LOQ), and correlation coefficient *R*^2^. The LOD was calculated as three times the standard deviation of the ultrapure water blank sign (BS) expressed as concentration divided by the slope of the analytical curve (C): LOD = 3 × BS/C, and the LOQ was obtained as ten times the standard deviation of the blank divided by the slope of the analytical curve: LOQ = 10 × BS/C. Table 2 shows the analytical parameters LOD, LOQ, and *R*^2^ obtained to determine minerals due to the erosion process. *R*^2^ values ranged from 0.9894 to 0.9994. The addition and recovery test results to verify the ICP OES method’s accuracy ranged from 94% to 102%. The values of *R*^2^ are as per the values established by the International Union of Pure and Applied Chemistry (IUPAC) [25]. According to Abbruzzini et al. [26], the digestion methods have an influence on the element recovery test. The method proposed in Section 4.5 was adequate in the recovery test.

A critical quantification in chemical analysis is the determination of detection limit (LOD), limit of quantification (LOQ), and correlation coefficient *R*^2^. The LOD was calculated as three times the standard deviation of the ultrapure water blank sign (BS) expressed as concentration divided by the slope of the analytical curve (C): LOD = 3*BS/C, and the LOQ was obtained as ten times the standard deviation of the blank divided by the slope of the analytical curve: LOQ = 10*BS/C. Table 2 shows the analytical parameters LOD, LOQ, and *R*^2^ obtained to determine minerals due to the erosion process. *R*^2^ values ranged from 0.9894 to 0.9994. The addition and recovery test results to verify the ICP OES method’s accuracy ranged from 94% to 102%. The values of *R*^2^ are as per the values established by the International Union of Pure and Applied Chemistry (IUPAC) [25]. According to Abbruzzini et al. [26], the digestion methods have an influence on the element recovery test. The method proposed in Section 4.5 was adequate in the recovery test.

### 2.3. Concentration Measures: Before and after the Exposure Time of the Erosive Challenge

**Figure 1:**
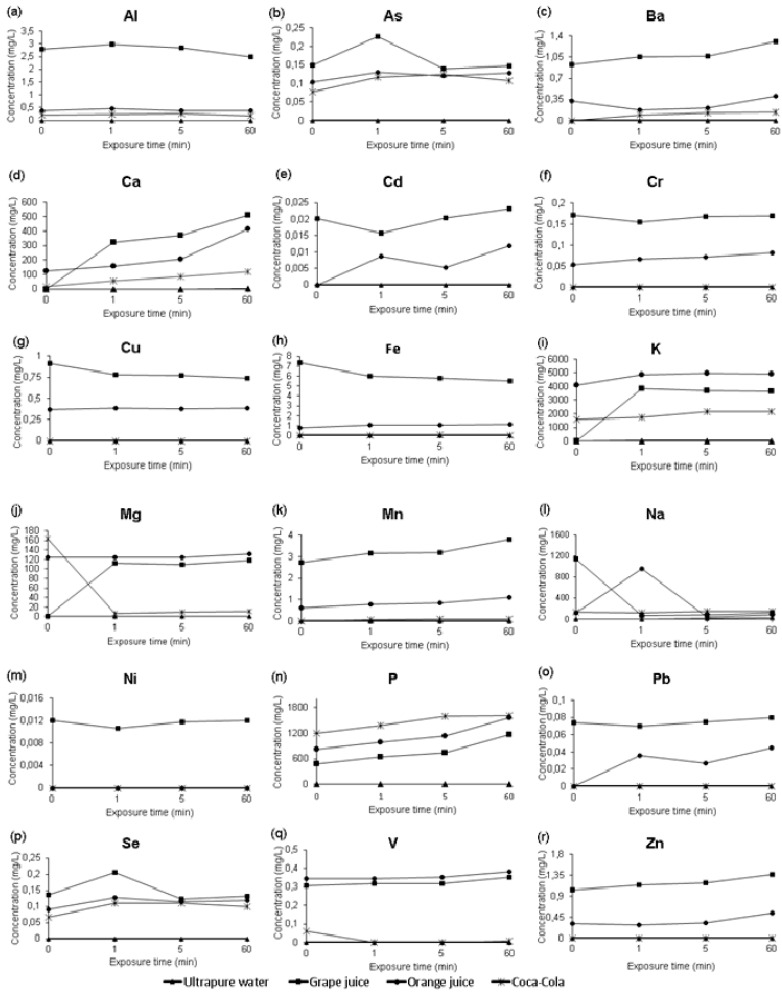
Elemental concentration in beverages from demineralization/remineralization process over time. (**a**) Al. (**b**) As. (**c**) Ba. (**d**) Ca. (**e**) Cd. (**f**) Cr. (**g**) Cu. (**h**) Fe. (**i**) K. (**j**) Mg. (**k**) Mn. (**l**) Na. (**m**) Ni. (**n**) P. (**o**) Pb. (**p**) Se. (**q**) V. (**r**) Zn.

The minerals’ concentration detected before (t = 0) and after the exposure time of the erosive challenge in vitro (t = 1, 5, and 60 min) and the ultrapure water results used as a control are shown in Table 3. A 2-way analysis of variance and a paired Tukey test were performed considering the results shown in Table 3. Figure 1 shows each chemical element’s behavior quantified in the drinks before and after the erosive challenge. Table 4 shows the results of the linear regression equation, coefficient of determination, and Pearson’s correlation coefficient obtained from the concentration of minerals detected in beverages before and after the erosive challenge. The linear regression shown in Table 4 comes from the data available in Table 3.

In the grape juice before the erosive challenge (t = 0) the following elements were quantified in decreasing order: Na > P > K > Fe > Al > Mn > Zn > Ba > Cu > V > Cr > Se > As > Pb > Cd > Ni, however elements such as Mg and Ca are below the detection limit (<LOD) (Table 3). On the other hand, the concentration of elements in grape juice for the exposure times of 1 min was K > P > Ca > Mg > Na > Fe > Mn > Zn > Ba > Cu > V > As > Se > Cr > Cd > Ni; for the exposure times 5 and 60 min were K > P > Ca > Mg > Na > Fe > Mn > Al > Zn > Ba > Cu > V > Cr > As > Se > Pb > Cd > Ni (Table 3). Some elements, such as Co and Mo, showed values below LOD. With the increase of the exposure time occurred an increase in the concentration of Na, P, K, Fe, Al, Mn, Zn, Ba, Cu, V, Cr, Se, As, Pb, Cd, and Ni in the grape juice, indicating that there was a process of demineralization of the teeth when immersed in grape juice (Figure 1). According to Table 4, there are very strong positive linear correlations between Al, Ba, Mn, P, V, and Zn in the grape juice and exposure time. Additionally, there are weak positive correlations for Cr, K; weak negative correlations for As, Na, Se; moderate positive correlations for Ca, Mg, Ni; strong positive correlations for Cd, Pb; and moderate negative correlations for Cu, Fe in the grape juice and exposure time.

For orange juice before the erosive challenge (t = 0) (Table 3), the elements were arranged as follows K > P > Na > Ca > Mg > Fe > Mn > Cu = Al > V > Ba > Zn > As > Se > Cr, however, elements such as Pb and Cd were below the LOD. By 1 min of exposure time, the elements decreased in the order: K > P > Na > Ca > Mg > Fe > Mn > Al > Cu > V > Zn > Ba > Se = As > Cr > Pb > Cd; at 5 min exposure time: K > P > Ca > Mg > Na > Fe > Mn > Al = Cu > V > Zn > Ba > As > Se > Cr > Pb > Cd; and at 60 min of exposure time: K > P > Ca > Mg > Na > Fe > Mn > Zn > Ba > V = Cu > Al > Se > As > Cr > Pb > Cd. The concentrations of Co, Mo and Ni in orange juice are below of LOD. It can be seen in Figure 1 that with the increase in the time of exposure of the teeth to orange juice, there was an increase in the values of the concentrations of elements. This means that due to the acidity of the orange juice there was a demineralization of the teeth. When comparing the concentration of elements between Mg, Mn, P, V, Zn in the orange juice and exposure time, the r-value correlations observed suggest a very strong positive correlation (Table 4), while a weak negative correlation was observed for Al, Na and exposure time. In addition, the concentration of Ba, Cd, Cr possibly had a strong strong positive correlation with exposure time. There is a weak positive correlation between K, Se, as well as a moderate negative correlation with the exposure time.

Before the exposure time, the elements quantified in Coca-Cola™ in decreasing order were: K > P > Mg > Na > Ca > Al > As > Se > V > F. However, Mn, Zn, Cu, Ba, Pb, Cd, Co, Cr, Mo, and Ni are below the detection limit (< LOD). On the other hand, after the erosive test the following elements were quantified in the exposure period t = 1: K > P > Na > Ca > Mg > Al > As > Se > Fe = Mn; for exposure time of 5 min: K > P > Na > Ca > Mg > Al > Ba > As > Se > Fe > Mn; and for the exposure time 60 min: K > P > Na > Ca > Mg > Al > Ba > Mn = As > Se > Fe. There is an variation in each element’s concentration in Coca-Cola™, depending on the exposure period (Figure 1). The results in Table 4 show that for the concentration of elements in Coca-Cola™ and exposure time, there are weak negative (Mg and V), moderate positive (Ba, Mn, P, Na and K), strong positive (Ca), weak positive (Se), strong negative (Al), and negligible positive correlations.

The finding in Table 4 indicates that the concentration of Ca, K, Mg, P, Na in ultrapure water and the time of exposure have a very strong positive correlation.

The 2-way ANOVA confirms that beverage type was critical for teeth demineralization in all elements, being accountable for a variation from 53.88% in calcium to 99.49% in nickel (Table 3). The second most important variable determining mineral concentration is the interaction between beverage type and time, except calcium. Time counted for 24.31% of the elemental content variation, being the second most important feature.

The linear equation shown in Table 4 represents the relationship between the concentration of minerals detected before and after the erosive challenge. As noted by Jager et al. [11], the regression lines of several drinks do not cross the *Y*-axis at or near the 0-level, indicating relatively high erosion during the first few minutes.

Notably, some calculated results show a positive (negative) correlation of element concentration with time exposure. In fact, a positive correlation was expected for calcium, as well as phosphurus, potassium, zinc, and copper, since it is the major enamel component [27].

## 3. Discussion

There was a difference in the pH values before and after exposure to the beverages. The pH value of Coca-Cola™ is lower than orange juice and grape juices. The pH value (Table 1) for grape juice is compatible with those found in other studies (pH between 3.0 and 4.0) [28]. Also, the pH values for Coca-Cola™ and orange juices in Table 1 are close to those obtained by Zimmer et al. [14], 2.47 and 3.87, respectively.

The results regarding orange juice and Coca-Cola™ pH are according to the obtained by Grobler et al. [22], with orange juice pH levels almost stable and Cola soda with increasing pH. Additionally, Jensdottir et al. [29] found that orange juice erosive potential is higher during the first three minutes of exposure and after 30 min, which perfectly fits our findings. The same study found that cola drinks’ erosive potential is more substantial during the first minutes, decreasing over 40-fold after three minutes, which explains the first pH peak rise and then its slowing down over time. Although the pH changed, in all drinks, the pH values remained below 5.0, described as the critical pH to cause tooth demineralization [11]. However, the literature may contain findings that contradict one another. According to Zimmer et al. [14], the pH alone gives no valid information about the erosivity of drinks. That is, enamel may be dissolved at a pH of 5.2–5.9 [30] and dentine at pH 6.0–6.8 [31]. In the present study (Table 1), the pH of ultrapure water remained constant during the erosion challenge (pH = 6.0), however, there was an increase in the concentration of the elements as a function of the exposure time (Table 3).

Grape juices contain acids such as tartaric, malic, and citric [32]. Compared to other drinks such as orange juice and Coca-Cola™, grape juice had the most significant demineralizing potential (Table 3) among the drinks studied, causing the loss of K, Ca, and Mg mainly. In fact, grape juices are more erosive than orange juices [15]. Studies using microhardness and loss of enamel structure have shown that bovine enamel specimens immersed in grape juice for 10 min at 37 °C, 3 times/day for 7 days, suffer erosion [15]. Compared to other drinks, orange juice caused a more substantial loss of K from the tooth (Figure 1). Orange juices have acids such as ascorbic acid and citric acid [33]. Beltrame et al. [15], analyzing microhardness, proved that bovine enamel immersed in orange juices for 10 min, 3 times/day for 7 days, also caused a loss of enamel structure.

Compared to other drinks, Coca-Cola™ caused a more significant loss of P from the tooth (Figure 1). Lutovac et al. [34], examining the enamel surface with Atomic Force Microscopy (AFM), observed enamel surface structure and microhardness changes after an exposure time of 5 min in Coca-Cola™. According to Yuan et al. [35], using an in situ study, the percentages of surface microhardness change on each exposure time (four days) due to demineralization. A model of an erosive challenge using Coca-Cola™ and exposure time from 3 to 30 min, and using atomic absorption spectroscopy, showed that Ca concentration in Coca-Cola™ depends linearly on exposure time [11].

Although pH values were lower in Coca-Cola™ than grape juice and orange juice (Table 1), the grape juice caused more significant damage to the teeth (measured by demineralization, according to Table 3). The orange juice caused a minor demineralization degree, and it can be explained once its pH is close to the critical pH of 5 to cause damage to teeth [11]. On the other hand, even Coca-Cola has a lower pH than grape juice, the immersion of teeth in it resulted in a lower demineralization than grape juice. That might have happened once the erosion depends not only on pH values that disregard undissociated acid but also on other factors such as titratable acidity (TA) [36]. Actually, energy drinks have the potential to promote mineral loss on the dental enamel surface due to the low pH (from 2.1 to 3.2) and high titratable acidity [3]; grape juice presented a higher TA than Cola soft drink in a study conducted by Beltrame et al. [15].

Time was the least important feature regarding the final mineral content in the solutions for all other elements. The demineralization process, set by liquids intake, would depend more on the frequency of consumption would cause more harm than the time of exposure itself once liquids do not stay in the mouth for a long time [37]. Nevertheless, time should be considered once there was a positive correlation between exposure time and the demineralization process (Table 4). In fact, there is a prevalence of dental erosion in adolescent competitive swimmers exposed to the neutral pH of the pool water; in this case, factors that increase the risk of dental erosion include the duration of swimming and the amount of training [38].

Despite undergoing demineralization in the presence of acidic liquids, the teeth can also absorb chemical elements from food [39], thus occurring a process of remineralization [40]. According to the results presented in Table 3 (Figure 1), at an exposure time of 1 min, the concentration of elements such as Ba (orange juice), Cd and Cr (grape juice), Mg (Coca-Cola™), Na (grape juice), Fe and Cu (grape juice) suffered a decrease after an erosive test, which can be explained simply by the absorption of these elements by the tooth.

Results obtained by Fujii et al. [9], Jager et al. [11], and Zimmer et al. [14] corroborate with our data (Table 3, Figure 1, and Table 4), so there is a positive correlation between demineralization and short exposure times. In fact, in all the papers that carried out the erosive challenge considering acid drinks [9,11,22], the demineralization due to loss of Ca occurs in the first minutes of tooth immersion in beverages. Exposure times from 1 to 60 min [9,11,22] result in very diverse interpretations and estimates an erosive potential. As Jager et al. [11] observed, other exposure times (3–30 min) generate different teeth demineralization variations. However, the experimental model proposed in Section 4.3 provides results consistent with those obtained in Fujii et al. [9], Jager et al. [11], and Zimmer et al. [14].

Although we did not consider an exposure time of 30 min in our experimental model (Table 4), it is sugested that the concentrations of some elements in grape juice, orange juice, and Coca-Cola™ are positively related to the exposure time. Mathew et al. [41] showed that different drinks have erosive potential on teeth depending on exposure time duration. We hypothesize that saturation occurs only for some chemical elements and occurs mainly over long periods of exposure. However, as Barbour et al. [12] stated, the degree of saturation probably has a non-linear relationship with erosion.

The saturation process of some elements such as Ca is a complex phenomenon and involves hydroxyapatite Ca_10_(PO_4_)_6_(OH)_2_. According to Puy [42], for a pH below 5.5, hydroxyapatite (HA) begins to release phosphate to balance the pH. Hydroxyapatite may have other minerals incorporated in its structure, mainly from the diet [34], such as Al, Cd, Co, Cu, Fe, K, Mg, Mn, S, Zn, etc. in which be present in the enamel and dentin molecules [16]. Although we use bovine teeth (Table 3), elements such as Al, As, Ba, Ca, Cd, Cr, Cu, Fe, K, Mg, Mn, Na, Ni, P, Pb, Se, V, and Zn are also present in human teeth [20,21].

According to Jameel et al. [2], Lussi et al. [5], Fujii et al. [9], Barbour et al. [12], one of the dominant factors in erosion is pH. Orange juice caused more significant losses of calcium, and Coca-Cola™ proved greater losses of phosphorus. Concerning other elements, losses were principally driven by the grape juice. Grape juice showed a higher erosive power than Coca-Cola™ and orange juice, probably due to its higher acidity. According to Beltrame et al. [15], grape juices presented a more considerable erosive potential than orange juices.

The action of saliva is known to considerably reduce the loss of tooth structure by erosion [43]. However, in our experiment, it was not possible to verify whether saliva reduced the loss of elements present in the teeth. According to Hannas et al. [44], there are several difficulties in reproducing oral conditions in in vitro studies due to the presence of the acquired pellicle, dynamic salivary flow, and bacteria, as well as temperature variations. However, although the results obtained in our models are valid within the experimental conditions only, factors such as control of temperature, salivary flow, agitation, pH, short and long exposure time, etc., need to be considered in new studies to obtain a better understanding of remineralization and demineralization processes and their effects on teeth. According to Alencar et al. [45], considering the reparative effect of human saliva, two hours of human salivary exposure seems appropriate for changes of the softened enamel surface between erosive challenges.

## 4. Materials and Methods

### 4.1. Teeth Selection

This study was conducted at the Federal University of Mato Grosso do Sul, School of Medicine, Brazil. A total of 240 bovine incisor teeth donated by Frigorífico Naturafrig Rochedo—MS were used in this research. After the animals were slaughtered for meat’s commercial purpose, the teeth were extracted and washed thoroughly under ultrapure water to eliminate saliva, blood, and tissue debris [20]. Calculus was removed with the aid of ultrasound (Dabi Atlante, Ribeirão Preto–SP, Brazil) and Gracey curettes. Thus, this project does not need the authorization of the ethics committee on animal research.

### 4.2. Purchase of Drinks

Five batches of Coca-Cola™ (Coca-Cola™ FEMSA, Campo Grande–MS, Brazil), red grape juice (Vinícola Aurora, Bento Gonçalves–RS, Brazil), and orange juice (Prats, Paranavaí–PR, Brazil) were purchased from 10 randomly selected supermarkets in Campo Grande, MS, Brazil, from August to December 2020.

### 4.3. Method on the Erosive Challenge In Vitro

A total of 80 samples of bovine incisor teeth were allocated into nine groups (n = 4) with an average of 22 ± 1.0 g. 150 mL of acidic beverages (orange juice, Coca-Cola™, and grape juice) and ultrapure water (18 MΩcm, control group) were used in their traditional forms and added to each group of teeth. The erosive challenge was carried out in triplicate, using a total of 240 teeth.

This research’s exposure time was based on the paper published by Fujii et al. [9]. The erosive challenges methodology was based on the mineral content measured before and after immersion of each group (22 ± 1.0 g) in 150 mL of chosen drinks. Therefore, the groups of bovine incisor teeth were submitted to the erosive challenge as follows:

(1) Demineralization by immersing the teeth in acidic drinks (orange juice, Coca-Cola™ and grape juice) and ultrapure water for 1 min, at 25 °C, without stirring; and then rinse in ultrapure water for 5 s;
(2) Remineralization by immersion in artificial saliva for 40 min, at 25 °C, without stirring; and then rinse in ultrapure water for 5 s, at 25 °C;
(3) Demineralization by immersion of teeth in acidic drinks (orange juice, Coca-Cola™, grape juice) and ultrapure water for 5 min, at 25 °C, without stirring; and then rinse in ultrapure water for 5 s, at 25 °C;
(4) Remineralization by immersion in artificial saliva for 40 min, at 25 °C, without stirring; and then rinse in ultrapure water for 5 s;
(5) Demineralization by immersing the teeth in acidic drinks (orange juice, Coca-Cola™, grape juice) and ultrapure water for 60 min, at 25 °C, without stirring.
(6) All the procedures described above were also done in ultrapure water used as a control group.

The artificial saliva (KinHidrat, PharmaKin, São Paulo–SP,) (pH 6.0) used for the remineralization of teeth is composed of the following reagents: potassium thiocyanate, potassium chloride, sodium chloride, calcium chloride, magnesium chloride, potassium dihydrogen phosphate, xylitol, sodium saccharin, sodium nipase-2, 2-nitropopane-1,3-diol, menthol, aroma, citric acid, hydrogenated castor oil, PEG 40 and purified water.

### 4.4. Monitoring of the pH

The pH and temperature of the acidic drinks were determined with a pH meter, model Q 402M (Quimis, Diadema–SP, Brazil). The temperature in the laboratory was 25 °C (±2 °C is expected). The pH measurements were taken before and after the teeth erosive challenge test in acidic drinks at different exposure times.

### 4.5. Microwave-Assisted Acid Digestion

Acid digestion is one of the most critical sample preparation techniques. 6 mL of each acidic drink (orange juice, Coca-Cola™, and grape juice), as well as 6 mL of the control group (ultrapure water), were collected before the erosive challenge (t = 0) and in the exposure periods of 1, 5, and 60 min. Subsequently, 2 mL of HNO_3_ (65%, Merck, Darmstadt, Germany) and 1 mL of H_2_O_2_ (30%, Merck, Darmstadt, Germany) were added to each sample in microwave digestion Teflon tubes (Speedwave four, Berghof, Eningen–BW, Germany). The following operating procedures for the microwave digestion were used: step 1 (100 °C for 5 min; pressure of 30 bar and 1.305 W of power); step 2 (150 °C for 10 min, pressure of 30 bar, and 1.305 W of power); step 3 (50 °C for 1 min, pressure of 25 bar and 1.305 W of power). After the procedure of digestion and subsequent cooling at room temperature, the digested samples were diluted with ultrapure water to a final volume of 10 mL. The digestion procedure was carried out in triplicate. The ultrapure water was used to run blanks (prepared with the addition of nitric acid, hydrogen peroxide and ultrapure water). The final acid concentration of the standards was approximately 13% of the acid blend to match the ultrapure water blank. Furthermore, an ultrapure water blank digest was carried out using the same digestion conditions for a microwave system.

### 4.6. Elemental Analysis Using ICP OES Technique

The determination of 20 elements in acidic drinks in the set times was carried out by ICP OES (iCAP 6300 Series, Thermo Scientific, Cambridge, UK). The instrumental setting and operational conditions were as following: a sample flush time of 30 s, pump stabilization time of 5.0 s, nebulizer gas flow of 0.7 L/min, the auxiliary gas flow of 0.5 L/min, flush pump rate of 50 rpm, radiofrequency power of 1150 W, analysis pump rate of 50 rpm, plasma view axial and a coolant gas flow of 12 L/min. The following analytical lines were used for each element: K 766.490 nm, P 177.495 nm, Ca 393.366 nm, Mg 279.553 nm, Mn 257.610 nm, Zn 213.856 nm, Fe 259.940 nm, Cu 324.754 nm, Ba 455.403 nm, Pb 220.353 nm, As 189.042 nm, Cd 228.802 nm, Al 167.079 nm, Co 228.616 nm, Cr 283.563 nm, Mo 202.030 nm, Na 588.995 nm, Ni 221.647 nm, Se 196.090 nm and V 309.311 nm.

For instrumental calibration, the 0.01, 0.025, 0.05, 0.1, 0.25, 0.5, 1.0, 2.0 and 4.0 mg/L intermediate standard solutions of each element was prepared by diluting a 100 mg/L stock standard solution. All experiments were performed in triplicate.

The limits of detection (LOD) and quantification (LOQ) were calculated according to Long and Winefordner [46]. An addition/recovery test was carried out to evaluate the accuracy of the standard internal method with the addition of 1 ppm of each element [47].

### 4.7. Statistical Analysis

Statistical analysis was performed with Origin version 8.1 (OriginLab Corporation, Northampton, MA, USA) for linear regression. Linear regression analysis was performed to determine the coefficient of determination (*R*^2^), which gives the percentage variation in y (concentration of elements) explained by x-variables (exposure times). That is, the *R*^2^ range is 0 to 1 (i.e., 0% to 100% of the variation in y can be explained by the x-variables) [48]. In addition, Pearson’s correlation coefficient (r) was calculated as a measure between the concentration of elements in the drinks and the exposure time (0, 1, 5, and 60 min). The “*r*” ranges from −1 to 1. We adopted the values of “cutoff of *r*” according to Schober et al. (2018) [48]; if the correlation coefficient between concentration of elements in the drinks and the exposure time range from 0.90 to 1.0 (−0.90 and −1.0), then this reveals a very strong positive (negative) linear correlation between the two variables; values between 0.70 and 0.89 (−0.70 and −0.89) indicate a strong positive (negative) linear correlation; values between 0.40 and 0.69 (−0.40 and −0.69) indicate a moderate positive (negative) correlation; values between 0.10 and 0.39 (−0.10 and −0.39) indicate a weak positive (negative) correlation and values between 0.00 and 0.10 (0.00 and −0.10) indicate a negligible positive (negative) correlation. In addition, a 2-way analysis of variance test (2-way ANOVA) was performed, followed by Tukey’s post hoc test to determine whether the variations derive from the beverage itself or from the time exposure; significant differences were considered when the p-value was below 0.05.

## 5. Conclusions

Considering the limitations of the present study, the proposed experimental model showed effectiveness in monitoring bovine teeth’ demineralization when immersed in acidic liquids such as grape juice, orange juice, and Coca-Cola™. For the first time, the concentration of elements such as Al, As, Ba, Ca, Cd, Cr, Cu, Fe, K, Mg, Mn, Na, Ni, P, Pb, Se, V, and Zn released from the bovine incisors was monitored during the erosive challenge in vitro at different times of exposure. There is a positive relationship between concentrations of the quantified elements in the drinks and time exposure.

The pH values varied slightly in all samples during the erosive challenge, although remaining below the critical pH of 5 to cause tooth demineralization. According to the comparisons, significant differences in the concentration of elements were detected among various drinks. The concentration of elements quantified in the drinks revealed grape juice has more significant erosive potential than Coca-Cola™ and orange juice.

In recent years, little attention has been given to the monitoring and quantifying chemical elements present in human or bovine teeth and saliva. Although some elements can protect teeth, they also can be toxic or cause dental damage; therefore, monitoring these elements is necessary.

The results obtained in this paper using bovine teeth open doors for further development studies considering other drinks, evaluation, consolidation, and validation methods.

## Figures and Tables

**Table 1:** Measurements at pH values and temperatures according to each drink’s exposure time before (t = 0) and after the erosive challenge (1, 5, 30, and 60 min).

Drink	Time (min)	pH	Temperature (°C)
Ultrapure water	0	6.0	26.1
	1	6.0	25.0
	5	6.0	26.0
	30	6.0	25.0
	60	6.0	26.8
Grape juice	0	3.57	25.9
	1	3.70	26.9
	5	3.73	27.2
	30	3.57	26.8
	60	3.81	26.9
Orange juice	0	4.50	22.0
	1	4.46	24.6
	5	4.47	24.4
	30	4.45	25.0
	60	4.47	26.4
Coca-Cola™	0	2.57	26.5
	1	3.12	26.4
	5	3.14	26.3
	30	3.35	26.6
	60	3.49	26.5

**Table 2:** Analytical data obtained by the ICP OES: limit of detection (LOD), limit of quantification (LOQ), correlation coefficient (*R*^2^), and percentage of recovery (%).

Elements	LOD (mg/L)	LOQ (mg/L)	(*R*^2^)	Recovery (%)
Al	0.0633	0.2110	0.9989	99
As	0.0056	0.0185	0.9994	98
Ba	0.0008	0.0026	0.9986	101
Ca	0.0041	0.0138	0.9981	100
Cd	0.0057	0.0192	0.9994	99
Co	0.0023	0.0076	0.9994	100
Cr	0.0115	0.0384	0.9994	102
Cu	0.0062	0.0207	0.9990	98
Fe	0.0036	0.0120	0.9994	97
K	0.0349	0.1163	0.9963	95
Mg	0.0015	0.0049	0.9987	94
Mn	0.0004	0.0014	0.9993	98
Mo	0.0016	0.0054	0.9994	100
Na	0.0029	0.0098	0.9960	99
Ni	0.0017	0.0058	0.9994	97
P	0.0811	0.2705	0.9894	99
Pb	0.0115	0.0385	0.9994	98
Se	0.0079	0.0263	0.9994	101
V	0.0019	0.0063	0.9993	99
Zn	0.0008	0.0026	0.9994	94

**Table 3:** Concentration of minerals detected before (t = 0) and after the erosive challenge in vitro (t = 1, 5, and 60 min). Values are expressed as mean ± SD. Statistical results: *p*-values.

Drinks	Exposure Time (min)	Elements (mg/L)									
		Al	As	Ba	Ca	Cd	Co	Cr	Cu	Fe	K
Ultrapure Water (Control)	**0**	<LOD	<LOD	<LOD	<LOD	<LOD	<LOD	<LOD	<LOD	<LOD	<LOD
	**1**	<LOD	<LOD	<LOD	<LOD	<LOD	<LOD	<LOD	<LOD	<LOD	0.14 ± 0.02
	**5**	<LOD	<LOD	<LOD	0.157 ± 0.004	<LOD	<LOD	<LOD	<LOD	<LOD	0.28 ± 0.02
	**60**	<LOD	<LOD	<LOD	0.80 ± 0.008	<LOD	<LOD	<LOD	<LOD	<LOD	0.671 ± 0.006
Grape Juice	**0**	2.79 ± 0.04	0.148 ± 0.004	0.929 ± 0.009	<LOD	0.020 ± 0.003	<LOD	0.172 ± 0.001	0.908 ± 0.009	7.34 ± 0.05	28.22 ± 0.65
	**1**	2.99 ± 0.07	0.226 ± 0.003	1.05 ± 0.02	322.1 ± 9.1	0.016 ± 0.003	<LOD	0.155 ± 0.006	0.78 ± 0.01	6.00 ± 0.07	3852.9 ± 61.7
	**5**	2.85 ± 0.03	0.139 ± 0.002	1.062 ± 0.008	372.6 ± 6.2	0.020 ± 0.006	<LOD	0.168 ± 0.004	0.769 ± 0.006	5.80 ± 0.02	3664.02 ± 115.94
	**60**	2.48 ± 0.03	0.146 ± 0.001	1.31 ± 0.04	513.79 ± 5.07	0.023 ± 0.001	<LOD	0.171 ± 0.003	0.742 ± 0.006	5.51 ± 0.02	3643.8 ± 105.4
Orange Juice	**0**	0.37 ± 0.03	0.103 ± 0.003	0.327 ± 0.007	125.4 ± 2.7	<LOD	<LOD	0.055 ± 0.003	0.371 ± 0.005	0.78 ± 0.01	4083.01 ± 63.45
	**1**	0.46 ± 0.03	0.130 ± 0.004	0.168 ± 0.002	156.5 ± 1.8	0.009 ± 0.003	<LOD	0.0683 ± 0.0005	0.380 ± 0.004	1.049 ± 0.008	4878.6 ± 75.0
	**5**	0.37 ± 0.03	0.122 ± 0.002	0.213 ± 0.005	204.57 ± 3.03	0.005 ± 0.002	<LOD	0.072 ± 0.003	0.373 ± 0.008	1.11 ± 0.01	4957.4 ± 97. 6
	**60**	0.37 ± 0.02	0.129 ± 0.001	0.402 ± 0.006	413.5 ± 1.9	0.0120 ± 0.0004	<LOD	0.0816 ± 0003	0.380 ± 0.005	1.131 ± 0.006	4920.8 ± 47.1
Coca-Cola™	**0**	0.21 ± 0.02	0.077 ± 0.004	<LD	17.7 ± 0.3	<LOD	<LOD	<LOD	<LOD	0.020 ± 0.001	1572.8 ± 31.2
	**1**	0.25 ± 0.02	0.117 ± 0.002	0.094 ± 0.001	55.7 ± 0.6	<LOD	<LOD	<LOD	<LOD	0.0514 ± 0.0003	1734.01 ± 60.54
	**5**	0.26 ± 0.03	0.125 ± 0.001	0.127 ± 0.003	84.1 ± 0.9	<LOD	<LOD	<LOD	<LOD	0.104 ± 0.003	2161.3 ± 34.8
	**60**	0.19 ± 0.02	0.107 ± 0.003	0.139 ± 0.006	119.1 ± 0.8	<LOD	<LOD	<LOD	<LOD	0.049 ± 0.005	2175.4 ± 81.9
† *p*-value			<0.0001	<0.0001	<0.0001	<0.01	ND	<0.001	<0.0001	<0.0001	<0.0001
**Drinks**	**Exposure Time (min)**	**Elements (mg/L)**									
		**Mg**	**Mn**	**Mo**	**Na**	**Ni**	**P**	**Pb**	**Se**	**V**	**Zn**
Ultrapure Water (Control)	**0**	<LOD	<LOD	<LOD	0.627 ± 0.002	<LOD	<LOD	<LOD	<LOD	<LOD	<LOD
	**1**	0.0343 ± 0.0006	<LOD	<LOD	4.72 ± 0.03	<LOD	0.538 ± 0.05	<LOD	<LOD	<LOD	<LOD
	**5**	0.256 ± 0.002	<LOD	<LOD	10.92 ± 0.04	<LOD	1.756 ± 008	<LOD	<LOD	<LOD	<LOD
	**60**	0.829 ± 0.004	<LOD	<LOD	27.0 ± 0.4	<LOD	4.70 ± 0.07	<LOD	<LOD	<LOD	<LOD
Grape Juice	**0**	<LOD	2.75 ± 0.02	<LOD	1147.5 ± 27.6	0.012 ± 0.001	476.8 ± 10.8	0.074 ± 0.008	0.136 ± 0.005	0.308 ± 0.002	1.038 ± 0.004
	**1**	112.0 ± 2.9	3.15 ± 0.04	<LOD	72.6 ± 0.5	0.011 ± 0.001	635.8 ± 14.4	0.070 ± 0.006	0.206 ± 0.003	0.318 ± 0.004	1.15 ± 0.01
	**5**	110.3 ± 1.0	3.196 ± 0.007	<LOD	81.6 ± 0.9	0.0118 ± 0.0007	744.7 ± 15. 6	0.075 ± 0.003	0.124 ± 0.002	0.318 ± 0.002	1.192 ± 0.003
	**60**	117.9 ± 0.4	3.828 ± 0.018	<LOD	123.4 ± 1.6	0.0121 ± 0.0005	1166.3 ± 23.6	0.080 ± 0.002	0.131 ± 0.002	0.351 ± 0.001	1.359 ± 0.007
Orange Juice	**0**	123.7 ± 1.3	0.624 ± 0.009	<LOD	127.0 ± 2.3	<LOD	817.15 ± 22.09	<LOD	0.091 ± 0.003	0.345 ± 0.004	0.311 ± 0.005
	**1**	123.9 ± 1.2	0.787 ± 0.006	<LOD	954.1 ± 26.2	<LOD	987.4 ± 25.6	0.035 ± 0.007	0.128 ± 0.003	0.345 ± 0.004	0.278 ± 0.003
	**5**	124.9 ± 0.2	0.860 ± 0.011	<LOD	47.09 ± 0.08	<LOD	1131.8 ± 12.6	0.027 ± 0.005	0.114 ± 0.003	0.352 ± 0.005	0.334 ± 0.001
	**60**	131.3 ± 0.8	1.098 ± 0.006	<LOD	77.10 ± 0.08	<LOD	1553.4 ± 22.7	0.044 ± 0.003	0.118 ± 0.002	0.381 ± 0.003	0.537 ± 0.003
Coca-Cola™	**0**	163.3 ± 2.4	<LOD	<LOD	131.0 ± 1.1	<LOD	1201.7 ± 28.3	<LOD	0.067 ± 0.004	0.0597 ± 0.0003	<LOD
	**1**	6.23 ± 0.01	0.0505 ± 0.0005	<LOD	126.3 ± 0.5	<LOD	1382.8 ± 13.0	<LOD	0.111 ± 0.004	<LOD	<LOD
	**5**	8.8 ± 0.03	0.089 ± 0.002	<LOD	148.8 ± 1.2	<LOD	1593.0 ± 15.6	<LOD	0.110 ± 0.002	<LOD	<LOD
	**60**	11.03 ± 0.06	0.107 ± 0.003	<LOD	147.5 ± 0.7	<LOD	1616.65 ± 2.08	<LOD	0.099 ± 0.002	0.008 ± 0.001	<LOD
† *p*-value		<0.0001	<0.0001	ND	<0.0001	ND	<0.0001	<0.0001	<0.0001	<0.0001	<0.0001

<LOD-analyte concentrations were below the limits of detection. SD (standard deviation). ND = not determined. † *p*-value considered significant when below 0.05 within columns.

**Table 4:** Regression equation, coefficient of determination and Pearson’s correlation coefficient obtained from the concentration of minerals detected before and after the erosive challenge.

Elements	Drinks	Regression Equation	Coefficient of Determination *R*^2^	Pearson’s Correlation Coefficient (r)	Interpretation (Correlation)
Al	Grape juice	y = −0.0068x + 2.8887	0.8346	−0.91	Very strong negative
	Orange juice	y = −0.0005x + 0.401	0.1258	−0.35	Weak negative
	Coca-Cola™	y = −0.0008x + 0.2388	0.4886	−0.70	Strong negative
	Ultrapure water	<LOD			
As	Grape juice	y = −0.0005x + 0.1724	0.1088	−0.33	Weak negative
	Orange juice	y = 0.0002x + 0.1176	0.2183	0.47	Moderate positive
	Coca-Cola™	y = 0.00005x + 0.1055	0.0042	0.07	Negligible positive
	Ultrapure water	<LOD			
Ba	Grape juice	y = 0.0051x + 1.0034	0.8822	0.94	Very strong positive
	Orange juice	y = 0.0028x + 0.2317	0.5729	0.76	Strong positive
	Coca-Cola™	y = 0.0012x + 0.0696	0.3270	0.57	Moderate positive
	Ultrapure water	<LOD			
Ca	Grape juice	y = 5.1525x + 217.12	0.4762	0.69	Moderate positive
	Orange juice	y = 4.3912x + 152.55	0.9674	0.98	Very strong positive
	Coca-Cola™	y = 1.2052x + 49.287	0.6645	0.82	Strong positive
	Ultrapure water	y = 0.0131x + 0.0243	0.9855	0.99	Very strong positive
Cd	Grape juice	y = 0.00008x + 0.0186	0.5435	0.74	Strong positive
	Orange juice	y = 00001x + 0.0044	0.5230	0.72	Strong positive
	Coca-Cola™	<LOD			
	Ultrapure water	<LOD			
Co	Grape juice	<LOD			
	Orange juice	<LOD			
	Coca-Cola™	<LOD			
	Ultrapure water	<LOD			
Cr	Grape juice	y = 0.0001x + 0.1651	0.1287	0.36	Weak positive
	Orange juice	y = 0.0003x + 0.0643	0.5997	0.77	Strong positive
	Coca-Cola™	< LOD			
	Ultrapure water	< LOD			
Cu	Grape juice	y = −0.0014x + 0.823	0.3122	−0.56	Moderate negative
	Orange juice	y = 0.0001x + 0.3742	0.3332	0.58	Moderate positive
	Coca-Cola™	<LOD			
	Ultrapure water	<LOD			
Fe	Grape juice	y = −0.0162x + 6.431	0.3381	−0.58	Moderate negative
	Orange juice	y = 0.0029x + 0.9691	0.2727	0.52	Moderate positive
	Coca-Cola™	<LOD			
	Ultrapure water	<LOD			
K	Grape juice	y = 22.146x + 2431.8	0.1214	0.35	Weak positive
	Orange juice	y = 5.5403x + 4618.5	0.1477	0.38	Weak positive
	Coca-Cola™	y = 6.6793x + 1800.7	0.4067	0.64	Moderate positive
	Ultrapure water	y = 0.0094x + 0.1195	0.8873	0.94	Very strong positive
Mg	Grape juice	y = 0.8371x + 71.224	0.1838	0.43	Moderate positive
	Orange juice	y = 0.9937x + 123.93	0.9937	1.00	Very strong positive
	Coca-Cola™	y = −0.9507x + 63.016	0.1279	−0.36	Weak negative
	Ultrapure water	y = 0.0128x + 0.0679	0.9496	0.97	Very strong positive
Mn	Grape juice	y = 0.014x + 2.9995	0.8349	0.91	Very strong positive
	Orange juice	y = 0.0061x + 0.7425	0.8013	0.90	Very strong positive
	Coca-Cola™	y = 0.0011x + 0.043	0.4782	0.69	Moderate positive
	Ultrapure water	<LOD			
Mo	Grape juice	<LOD			
	Orange juice	<LOD			
	Coca-Cola™	<LOD			
	Ultrapure water	<LOD			
Ni	Grape juice	y = 0.00001x + 0.0114	0.2227	0.47	Moderate positive
	Orange juice	<LOD			
	Coca-Cola™	<LOD			
	Ultrapure water	<LOD			
P	Grape juice	y = 9.636x + 596.9	0.9031	0.95	Very strong positive
	Orange juice	y = 10.157x + 954.86	0.8805	0.94	Very strong positive
	Coca-Cola™	y = 4.2341x + 1378.7	0.3977	0.63	Moderate positive
	Ultrapure water	y = 0.0693x + 0.6046	0.9218	0.96	Very strong positive
Pb	Grape juice	y = 0.0001x + 0.0725	0.7458	0.86	Strong positive
	Orange juice	y = 0.0004x + 0.0198	0.4089	0.64	Moderate positive
	Coca-Cola™	<LOD			
	Ultrapure water	<LOD			
Na	Grape juice	y = −6.1605x + 457.94	0.1151	−0.34	Weak negative
	Orange juice	y = −5.5473x + 392.88	0.1366	−0.37	Weak negative
	Coca-Cola™	y = 0.2305x + 134.61	0.3435	0.59	Moderate positive
	Ultrapure water	y = 0.3804x + 4.511	0.9117	0.95	Very strong positive
Se	Grape juice	y = −0.0005x + 0.157	0.1216	−0.35	Weak negative
	Orange juice	y = 0.0001x + 0.1106	0.0607	0.25	Weak positive
	Coca-Cola™	y = 0.00009x + 0.0953	0.0156	0.12	Weak positive
	Ultrapure water	<LOD			
V	Grape juice	y = 0.0006x + 0.3131	0.9609	0.98	Very strong positive
	Orange juice	y = 0.0006x + 0.3461	0.9850	0.99	Very strong positive
	Coca-Cola™	y = −0.0002x + 0.0211	0.0635	−0.25	Weak negative
	Ultrapure water	<LOD			
Zn	Grape juice	y = 0.0042x + 1.1151	0.8165	0.90	Very strong positive
	Orange juice	y = 0.004x + 0.2997	0.9757	0.99	Very strong positive
	Coca-Cola™	<LOD			
	Ultrapure water	<LOD			

<LOD-analyte concentrations were below the limits of detection.

## Data Availability

The data used to support the findings of this study are available from the corresponding author upon request.

## Sample Availability

Samples of the compounds are available from the authors.
